# FAM98 Family Proteins Play Distinct Roles in Osteoclastogenesis and Bone Resorption

**DOI:** 10.3390/biology14010045

**Published:** 2025-01-09

**Authors:** Lei Wang, Tarun Minocha, Bhaba K. Das, Mikaela D. Kunika, Aarthi Kannan, Ling Gao, Subburaman Mohan, Weirong Xing, Kottayil I. Varughese, Haibo Zhao

**Affiliations:** 1 Department of Orthopedics, The Third People’s Hospital of Hefei, Third Clinical College, Anhui Medical University, Hefei 230032, China; frankwl160902@163.com; 2 Southern California Institute for Research and Education, VA Long Beach Medical Center, Long Beach, CA 90822, USA; tarun.minocha@va.gov (T.M.); bhaba.das@va.gov (B.K.D.); mikaela.kunika@va.gov (M.D.K.); aarthi.kannan@va.gov (A.K.); ling.gao@va.gov (L.G.); kiypevarughese@gmail.com (K.I.V.); 3 Department of Dermatology, University of California-Irvine, Irvine, CA 92617, USA; 4 Musculoskeletal Disease Center, VA Loma Linda Healthcare System, Loma Linda, CA 92357, USA; subburaman.mohan@va.gov (S.M.); weirong.xing@va.gov (W.X.)

**Keywords:** Fam98a, Fam98b, Fam98c, osteoclast, Plekhm1, lysosome, bone resorption

## Abstract

There are three FAM98 family proteins (FAM98A/B/C) in humans and mice. Their physiological functions remain largely unknown. We have previously reported that Fam98a interacts with Plekhm1 in murine osteoclasts and functions in lysosome trafficking/secretion and bone resorption in osteoclasts in vitro. In this study, we found that all three *Fam98* genes were expressed in precursor and mature osteoclasts. While the knockdown of Fam98c by a specific short-hairpin RNA (shRNA) in osteoclast precursors attenuated osteoclastogenesis, depletion of Fam98b by an shRNA specifically disrupted osteoclast lysosome trafficking and bone resorption with phenotypes similar to Fam98a shRNA-knockdown in our previous study. Loss of Fam98a in myeloid osteoclast precursors was dispensable for trabecular and cortical bone mass in mice, as well as osteoclastogenesis/bone resorption in vitro, possibly due to compensation by increased Fam98b expression in Fam98a-null osteoclasts. These findings indicate that the three Fam98 proteins play distinct roles in osteoclastogenesis and osteoclast function and need further investigation in future studies.

## Simple Summary

In this study, we found that all three *Fam98* genes were expressed in precursor and mature osteoclasts. By applying in vitro and in vivo genetic approaches, we unveiled that Fam98c plays a critical role in osteoclast differentiation in vitro, whereas Fam98b was found to regulate osteoclast lysosome trafficking and bone resorption, as reported previously for Fam98a. Loss of Fam98a in myeloid osteoclast precursors was dispensable for trabecular and cortical bone homeostasis in mice, as well as osteoclastogenesis/bone resorption in vitro, possibly due to compensation by increased Fam98b expression. Based on these data, we predict that Fam98 proteins play distinct roles in osteoclastogenesis and osteoclast function.

## 1. Introduction

Osteoclasts play a pivotal role in skeleton growth/modeling, homeostasis/remodeling, and fracture repair [1]. The aberrant bone resorption due to either increased or diminished osteoclast number and/or activity is detrimental to skeleton health and causes anomalous bone mass and bone erosion in multiple bone diseases, including osteoporosis and osteopetrosis. Thus, unveiling the molecules and pathways regulating osteoclast differentiation and function under physiological and pathological conditions will not only advance our knowledge in osteoclast biology but also identify therapeutic targets for the treatment of developmental and degenerative bone diseases.

Osteoclast differentiation is regulated by two cytokines, M-CSF (macrophage colony-stimulating factor) and RANKL (a receptor activator of nuclear factor-kB ligand) [2]. Upon attachment of osteoclasts to bone matrix, the activating signals triggered by M-CSF and RANKL, as well as those transduced through the integrins and the immunoreceptors, act in concert to promote osteoclast actin cytoskeleton organization to form actin rings at the sealing zone that tightly seal the resorptive microenvironment. Subsequently, the protons and hydrolases are released via lysosome secretion to dissolve bone minerals and degrade bone matrix, respectively. Therefore, both cytoskeleton organization and lysosome secretory pathway are essential for osteoclast activation and bone resorption.

Of the key molecules regulating osteoclastogenesis and function identified so far, PLEKHM1 is specifically indispensable for lysosome trafficking and secretion and bone resorption in osteoclasts [3,4]. Mutations in the *PLEKHM1* gene cause an intermediate form of osteopetrosis in humans and in incisors absent (*ia*/*ia)* rats [5]. Moreover, both germline- and osteoclast-specific *Plekhm1* knockout mice have high bone mass phenotypes resulting from defective bone resorption [6]. PLEKHM1 regulates osteoclast lysosomal trafficking and formation of the ruffled border through its interaction with RAB7, a small GTPase that specifically regulates lysosomal pathways in eukaryotic and mammalian cells [7,8,9,10]. RAB7 is localized at the ruffled border in bone-resorbing osteoclasts. Depletion of Rab7 expression or disruption of Plekhm1-Rab7 interaction attenuates lysosome secretion and bone resorption in cultured rat and murine osteoclasts in vitro [11,12]. In addition to Rab7, we have previously identified several Plekhm1-binding proteins in murine osteoclasts including Fam98a (family with sequence similarity 98 member A) that may mediate PLEKHM1’s function in osteoclasts [6].

The FAM98 family proteins have three members in humans and mice (FAM98A, FAM98B, and FAM98C). Their physiological functions remain largely unknown. FAM98A has been reported as a substrate of protein arginine methyltransferase 1 (PRMT1) and may be involved in tumor cell migration and invasion [13]. It has been found to localize at stress granules upon various stress stimuli [14]. We have unveiled that Fam98a interacts with Plekhm1 in murine osteoclasts and knockdown of Fam98a expression by lentiviral transduction of specific short hairpin RNAs (shRNAs) disrupting lysosome trafficking/secretion and bone resorption in osteoclasts in vitro [6]. FAM98B has been identified as a component of tRNA ligase and RNA capping/transporting complexes regulating RNA metabolism and translation [15,16,17,18]. FAM98C has been suggested as a candidate gene regulating ciliogenesis, and the mutation of which may cause ciliopathies [19]. The co-expressions and interactions among FAM98A/B/C in mammalian cells and tissues have not been reported.

In this study, we investigated the roles of Fam98 family proteins in osteoclastogenesis and bone resorption by the generation of Fam98a myeloid conditional knockout mice and the depletion of Fam98b and Fam98c expression by lentiviral transduction of specific shRNAs in control and Fam98a-deficient bone marrow monocytes.

## 2. Materials and Methods

### 2.1. Reagents

The reagents used in this study were listed in Table 1.

### 2.2. Bone Marrow Monocyte and Osteoclast Cultures

Whole bone marrow cells were extracted from the tibias and femurs of 10-week-old mice. Bone marrow monocytes (BMMs) and osteoclasts were cultured as described previously. In brief, red blood cells were lysed in 1× red blood cell lysis buffer for 5 min at room temperature. Bone marrow cells were plated onto a 100 mm Petri dish and cultured in α-MEM containing 10% heat-inactivated FBS, 1× Penicillin–Streptomycin-L-Glutamine (PSG), and 1/10 volume of CMG 14–12 (conditioned medium supernatant containing recombinant M-CSF at 1 μg/mL) [20] for 4 days. To generate osteoclasts from BMMs, 1.5 × 10^4^ or 3 × 10^4^ BMMs were cultured in α-MEM culture medium with 1/100 volume of CMG 14–12 (equal to 10 ng/mL of M-CSF) and 100 ng/mL of recombinant RANKL for 4–5 days [6].

### 2.3. Tartrate-Resistant Acid Phosphatase (TRAP) Staining

BMMs cultured in a 48-well plate were fixed with 4% paraformaldehyde in phosphate-buffered saline (PBS) for 20 min at room temperature. TRAP was stained with NaK tartrate and Naphthol AS-BI phosphoric acid solution. The number of TRAP^+^ multinucleated osteoclasts with more than 3 nuclei/well in four wells was counted and analyzed by one-way analysis of variance (ANOVA).

### 2.4. Real-Time Reverse Transcription Quantitative Polymerase Chain Reaction (RT-qPCR)

Total RNAs were purified using an RNeasy mini kit, and the first strand cDNAs were generated from 1 μg of total RNA using the High-Capacity cDNA Reverse Transcription Kit following the manufacturer’s instructions. Real-time qPCR was performed using the specific primers and TaqMan Gene Expression Master Mix in the QuantStudio3 real-time PCR system (Thermo-Fisher Scientific, Waltham, MA, USA) with an initial denaturation at 95 °C for 10 min, followed by 40 cycles of 95 °C for 15 s and 60 °C for 1 min. The relative cDNA amount was calculated by normalizing to that of the mitochondrial gene *Mrps2* using the ΔCt method.

### 2.5. Lentiviral Transduction

The recombinant lentiviruses expressing shRNAs targeting the murine Fam98b, Fam98c, and firefly luciferase (LUC) were generated. The 293-T cells were plated at a density of 0.75 × 10^6^ cells/well in a 6-well tissue culture plate and cultured in DMEM medium containing 10% heat-inactivated FBS and 1 × PSG a day before transfection. The specific pLKO.1 gene transfer vectors (1.5 µg each) were co-transfected respectively with the 1.5 µg of lentivirus packing vectors, pCMV-delta-R8.2 and pMD2.G (at 8:1 ratio), using the TransIT-LT1 transfection reagent (9 µL in 100 µL of Opti-MEM/transfection). The medium was changed the next day. The virus supernatants were collected after 24 h and were filtrated through a 0.45 µm sterile nylon syringe filter (CAT# 76479-028, VWR, Radnor, PA, USA). For lentiviral transduction, 5 × 10^6^ bone marrow cells were cultured in a 100 mm Petri dish for 3 days. The cells were then transduced with virus supernatant and 20 µg/mL of Protamine for 24 h. The BMMs were then selected in α-MEM culture medium containing 6 µg/mL puromycin for 3 days before use.

### 2.6. Immunoblotting

Cells were lysed in RIPA buffer containing protease inhibitor cocktail. A total of 20 μg of proteins were separated by 8% SDS-polyacrylamide gel electrophoresis (PAGE). The proteins were detected with primary antibodies at 4 °C overnight, followed by incubation with HRP-conjugated anti-mouse secondary antibody. After rinsing three times with Tris-buffered saline containing 0.1% Tween 20, the membrane was subjected to enhanced chemiluminescent detection reagents used at 1:10 dilution.

### 2.7. Immunofluorescence

The immunofluorescent staining was performed as described in our earlier work [21]. The triple fluorescence-labeled osteoclasts were examined and analyzed using a Zeiss LSM 900 laser confocal scanning microscope run by Zeiss ZenBlue 3.1.

### 2.8. Resorption Pit Staining

The resorption pit staining and quantification were conducted as described in our previous publications [6,12].

### 2.9. Generation of Fam98a-Flox and Fam98a-Flox;LysM-Cre Mice

The *Fam98a*-flox mice on C57BL6/J background were commercially generated by Cyagen Biosciences. The *Lyz2*-Cre (LysM-Cre) mice on C57BL6/J background (B6.129P2^tm1(cre)Ifo^, CAT# 004781) were obtained from The Jackson Laboratory (Bar Harbor, ME, USA). The offspring of *Fam98a*-flox/+;LysM-Cre/Cre breeding pairs were genotyped for *Fam98a*-flox and *Lyz2*-Cre using primers listed in the above table and from The Jackson Laboratory. The animal work follows the ARRIVE guidelines. All the in vivo and in vitro experiments were performed and analyzed in a double-blinded manner.

### 2.10. Micro-CT

The femurs and L4 vertebrae isolated from 10-week-old male and female mice were cleaned of soft tissues and fixed in 4% paraformaldehyde in PBS for 3 days at 4 °C. The bones were imaged in a μCT (model vivaCT40; Scanco Medical AG, Wangen-Brüttisellen, Switzerland) with 55 to 70 kVp volts at a voxel size of 10.5 μm. A threshold of 200 was applied to all scans at medium resolution (E = 55 kVp, I = 145 μA, and integration time = 200 ms).

## 3. Results

### 3.1. All Three Murine Fam98 Family Genes Are Expressed in Precursor and Mature Osteoclasts

**Figure 1:**
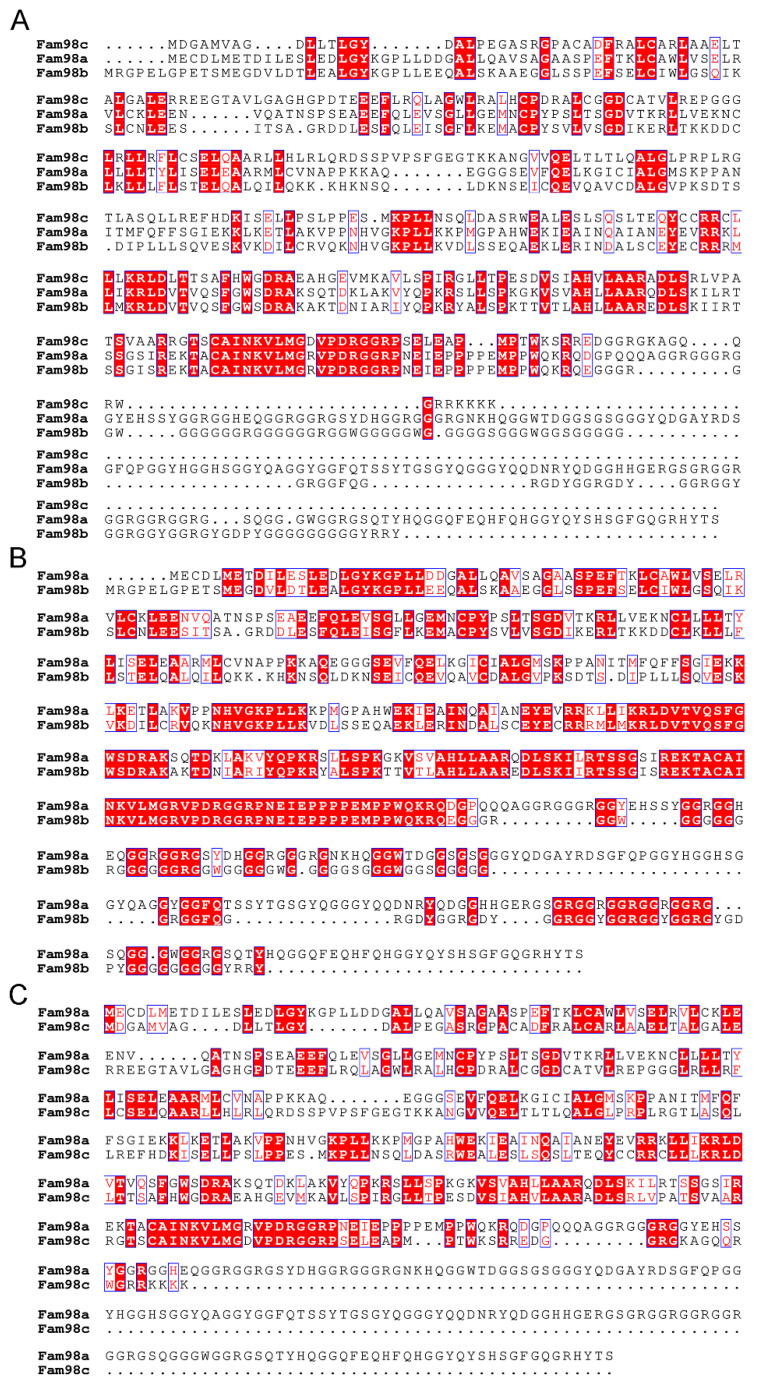
**Alignment of the three isoforms of murine Fam98 proteins generated by ClustaIW.** (**A**) Alignment of all three Fam98 family proteins. (**B**) Alignment between Fam98a and Fam98b. (**C**) Alignment between Fam98a and Fam98c. The open boxes with red letters show the highly conserved amino acids. The red-filled boxes with white letters illustrate the identical amino acids among the three isoforms of murine Fam98 proteins.

**Figure 2:**
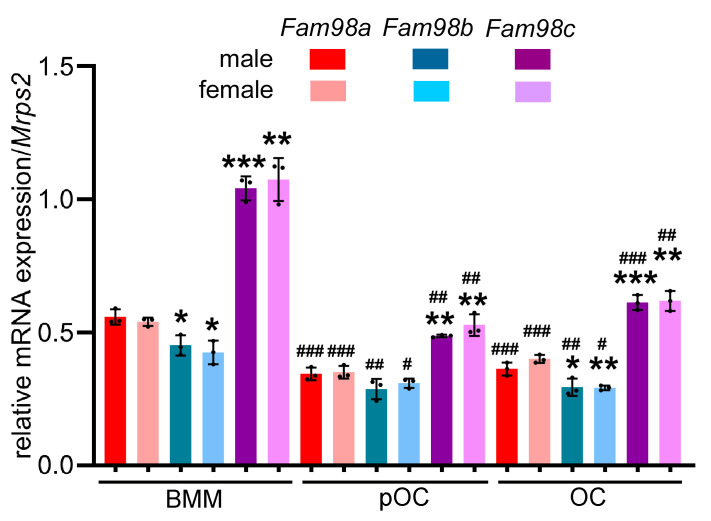
**The mRNA expression of three members of murine *Fam98* genes in male and female osteoclast precursor and mature cells.** The total RNAs were purified from three independent cultures of bone marrow monocytes (BMMs), mononuclear osteoclasts (pOCs), and multinucleated mature osteoclasts (OCs). The RNAs were reverse transcribed and quantified by real-time quantitative PCR (RT-qPCR). * *p* < 0.05, ** *p* < 0.01, *** *p* < 0.001 vs. respective *Fam98a*; ^#^ *p* < 0.05, ^##^ *p* < 0.01, ^###^ *p* < 0.001 vs. corresponding BMM analyzed by one-way ANOVA, *n* = 3.

In humans and mice, there are three FAM98 proteins (FAM98A, FAM98B, and FAM98C), which are encoded by distinct genes located on different chromosomes. FAM98A is the longest, and FAM98C is the shortest one among the three FAM98 proteins. The murine Fam98a has 19.6% identity and 22.7% similarity with both Fam98b and Fam98c (Figure 1). Fam98b is closer to Fam98a than Fam98c, with 46.3% identity and 55.0% similarity, while Fam98c has only 25% identity and 33.4% similarity with Fam98a. To dissect the roles of FAM98s in osteoclasts, we first examined their mRNA expression during in vitro osteoclast differentiation from murine bone marrow monocytes (BMMs) by real-time quantitative RT-PCR. As shown in Figure 2, the expression of *Fam98c* was found to be the highest among three genes in male and female osteoclast precursors and mature cells. The expression of *Fam98b* was slightly lower than that of *Fam98a* in osteoclast lineage cells. The mRNA expression of all three *Fam98* genes decreased during the differentiation of both male and female osteoclasts. The protein level of Fam98s in murine osteoclast lineage cells was not assessed in this study because of a lack of reliable antibodies specifically recognizing each isoform of Fam98 family proteins.

### 3.2. Knockdown of Fam98c but Not Fam98b Expression Attenuates Osteoclastogenesis In Vitro

**Figure 3:**
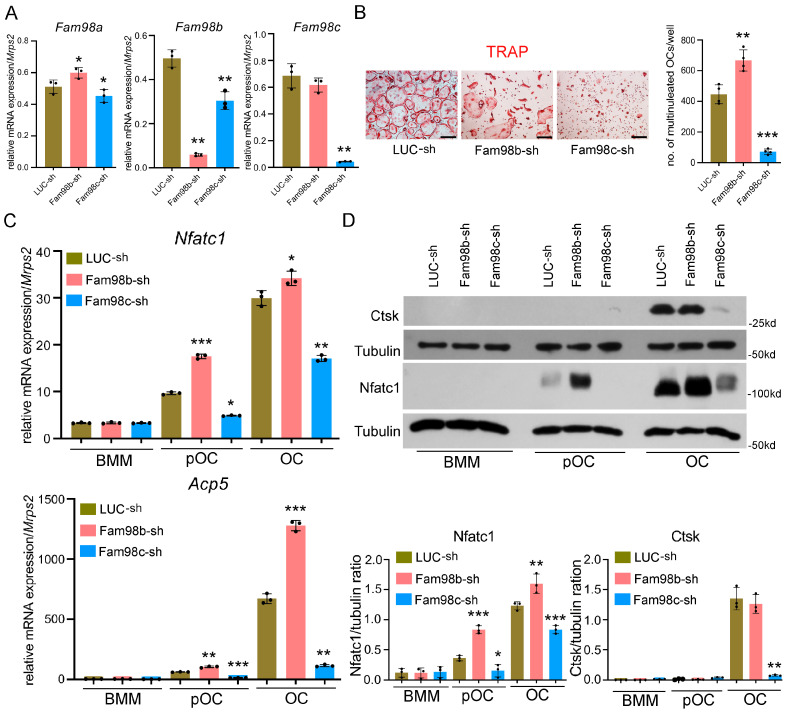
**Knockdown of Fam98c but not Fam98b expression in BMMs attenuates osteoclastogenesis in vitro.** (**A**) The real-time RT-qPCR detection of mRNA expression of *Fam98* isoforms in BMMs transduced with the lentiviruses expressing control shRNA against firefly luciferase (LUC-sh) and the specific shRNAs targeting *Fam98b* and *Fam98c*, respectively. (**B**) The TRAP (tartrate-resistant acid phosphatase) staining and cell number count of osteoclasts with more than 3 nuclei/cell. Scale bar = 40 µm. (**C**) The mRNA expression of osteoclast marker genes, *Nfatc1* and *Acp5*, in lentiviral transduced BMMs, pOCs, and OCs detected by RT-qPCR. (**D**) The protein expression of osteoclast markers, Nfatc1 and Cathepsin K (Ctsk), in lentiviral transduced BMMs, pOCs, and mature OCs probed by immunoblotting. The blot of tubulin served as a loading control (Supplementary Materials). * *p* < 0.05, ** *p* < 0.01, *** *p* < 0.001 vs. respective LUC-sh transduced cells analyzed by one-way ANOVA, *n* = 3–4.

We previously reported that Fam98a binds to Plekhm1, and the knockdown of Fam98a expression in vitro attenuates lysosome trafficking and secretion in murine osteoclasts [6]. However, whether and how Fam98b and Fam98c regulate osteoclasts remain unknown. To unveil the role of Fam98b and Fam98c in osteoclast differentiation and/or function, we set out to knock down their expression in BMMs by lentiviral transduction of specific shRNA targeting Fam98b (Fam98b-sh) and Fam98c (Fam98c-sh), respectively. The expression of Fam98b-sh significantly reduced the mRNA level of *Fam98b* and led to a slight increase in *Fam98a* mRNA in BMMs relative to BMMs transduced with the control shRNA against firefly luciferase (LUC-sh) (Figure 3A), whereas Fam98c-sh expression in BMMs robustly decreased the amount of *Fam98c* mRNA but also resulted in a little decrease in *Fam98a* and *Fam98b* mRNAs compared to control BMMs (Figure 3A).

We next cultured the different shRNA transduced BMMs with M-CSF and RANKL for 4 days to induce osteoclast differentiation. As shown by TRAP (tartrate-resistant acid phosphatase) staining and osteoclast number count (Figure 3B), the depletion of Fam98c greatly attenuated the formation of multinucleated osteoclasts (>3 nuclei/cell). Moreover, the mRNA and protein levels of Nfatc1, a master transcription factor of osteoclast differentiation, and the mRNA expression of osteoclast marker gene *Acp5* (encodes TRAP), as well as the protein level of Cathepsin K, a crucial bone matrix-digesting enzyme in mature osteoclasts, decreased dramatically in Fam98c-suppressed osteoclast precursor and mature cells (Figure 3C,D). This result indicates that Fam98c is critical for osteoclastogenesis, probably because it regulates Nfatc1 expression in osteoclast lineage cells. By contrast, the repression of Fam98b expression in osteoclast precursors caused an increase in osteoclast number, as affirmed by higher levels of Nfatc1 and Acp5 in mononuclear and mature osteoclasts compared to control cells (Figure 3B–D). Of note, however, about 50% of Fam98b-knockdown osteoclasts (50.7 ± 8.4%, *n* = 4) displayed a similar morphological cell shape to Plekhm1^−/−^ osteoclasts with aggregated TRAP^+^ lysosomes surrounding nuclei (Figure 3B, middle panel), suggesting that Fam98b, like Fam98a, may regulate osteoclast lysosome trafficking/secretion and bone resorption.

### 3.3. Decreased Expression of Fam98b in Osteoclasts Inhibits Lysosome Trafficking and Bone Resorption In Vitro

**Figure 4:**
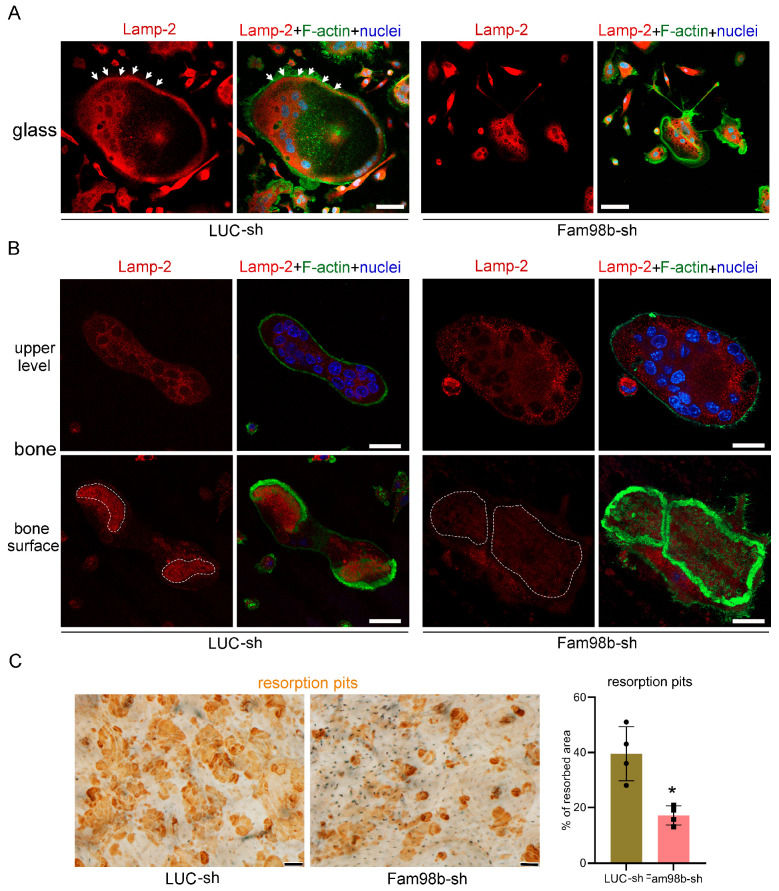
**Decreased expression of Fam98b in osteoclasts inhibits lysosome trafficking and bone resorption in vitro.** (**A**) The immunofluorescent staining of lysosome membrane protein Lamp-2 in control (LUC-sh) and Fam98b knockdown (Fam98b-sh) osteoclasts cultured on glass coverslips. The filament actin (F-actin) and nuclei were stained with the Alexa-488 conjugated phalloidin and Hoechst-33342, respectively. The white arrows point to the peripheral distributed Lamp-2 in control osteoclasts. Scale bar = 30 µm. (**B**) The immunofluorescent staining of Lamp-2 in control and Fam98b knockdown osteoclasts cultured on bovine cortical bone chips. The white dashed lines demarcate the Lamp-2 staining at the ruffled border circumscribed by actin rings in control osteoclasts. Scale bar = 15 µm. (**C**) The resorption pits labeled by the horseradish peroxidase (HRP) conjugated wheat germ agglutinin (WGA) lectin were stained by 3,3′-diaminobenzidine (DAB). Scale bar = 10 µm. The percentage of resorbed area was quantified with the NIH ImageJ2 software and analyzed by Student’s *t*-test. * *p* < 0.05 vs. control osteoclasts. *n* = 4.

To further define the function of Fam98b in osteoclasts, we cultured the control (LUC-sh) and Fam98b-knockdown (Fam98b-sh) BMMs with M-CSF and RANKL for 4 days on glass coverslips and cortical bovine bone slices, respectively. Lysosomes were labeled with a rat monoclonal antibody against murine Lamp-2, an integral membrane protein and marker of lysosomes in mammalian cells [22], co-stained with filament actin (F-actin) and nucleus. While a significant number of Lamp-2^+^ lysosomes were observed to be transported to and localized at the peripheral area inside the podosome ring in control osteoclasts cultured on glass coverslips, the lysosomes in Fam98b-deficient osteoclasts were aggregated around nuclei, void from the cell periphery (Figure 4A). In control osteoclasts cultured on bone slices, the Lamp-2^+^ lysosomes were targeted to the ruffled border membrane circumscribed by actin rings (Figure 4B, left panels). However, the Lamp-2 staining in Fam98b-deficient osteoclasts was mostly absent at the ruffled border (Figure 4B, right panels). Consistent with the important role of lysosome secretion in osteoclast bone resorption, the defective lysosome transportation in Fam98b-depressed osteoclasts led to a 50% reduction in bone resorption compared to control osteoclasts (Figure 4C).

### 3.4. Fam98a and Fam98b Compensate in Regulation of Lysosome Secretion and Bone Resorption in Osteoclasts

**Figure 5:**
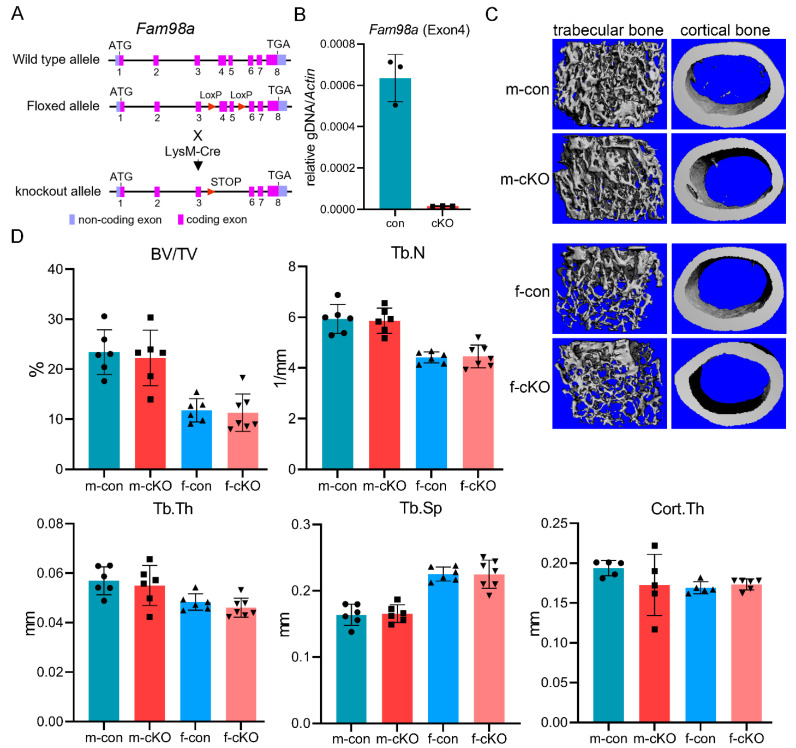
**Loss of Fam98a in myeloid osteoclast precursor cells is dispensable for bone mass and structure in mice.** (**A**) A cartoon illustration of the strategy to generate Fam98a conditional knockout (cKO) mice in LysM-Cre expressing myeloid osteoclast precursors. (**B**) The deletion of *Fam98a* exon 4 genomic DNA in BMMs isolated from Fam98a cKO mice detected by qPCR. *n* = 3. (**C**,**D**) The micro-CT images and analyses of trabecular and cortical bone compartments in distal femurs of 10-week-old male and female mice. *n* = 6–7.

To elucidate the role of FAM98A in the regulation of osteoclasts and bone homeostasis in vivo, we generated the *Fam98a*-flox mice in which the exons 4 and 5 of murine *Fam98a* gene were flanked by two loxP sites (Figure 5A). By crossing *Fam98a*-floxed mice with LysM-Cre mice, we created the Fam98a myeloid osteoclast precursor conditional knockout mice (cKO) (Figure 5A). The real-time quantitative PCR confirmed the deletion of exon 4 of the murine *Fam98a* gene in BMMs cultured from Fam98a cKO mice (Figure 5B). To maximize the deleting efficacy of Fam98a by LysM-Cre, we mated the *Fam98a*-flox/+; LysM-Cre/Cre breeding pairs to generate the control (Fam98a-+/+; LysM-Cre/Cre) and the Fam98a cKO (Fam98a-flox/flox;LysM-Cre/Cre) mice, similar to what we did in our recent published work [23]. Unlike severe human osteopetrosis patients and mouse models [24], Fam98a cKO mice displayed no overt phenotypes in growth, body weight, craniofacial growth and development, and tooth eruption until 10 weeks of age. The femurs and lumber vertebrae were harvested from 10-week-old male and female mice for micro-CT analysis of bone mass and structures. As presented in Figure 5C,D of micro-CT images and analyses, the trabecular bone volume (BV/TV) and cortical bone thickness (Cort.Th) in distal femurs of Fam98a cKO male and female mice were similar to those of control mice. The trabecular number (Tb.N), thickness (Tb.Th), and trabecular spacing (Tb.Sp) in Fam98a cKO male and female mice were indistinguishable from those of control mice. Additionally, there were no changes in the skeletal phenotypes of lumber vertebra in Fam98a cKO mice. These results indicate that loss of Fam98a in osteoclasts is dispensable for bone homeostasis in vivo.

**Figure 6:**
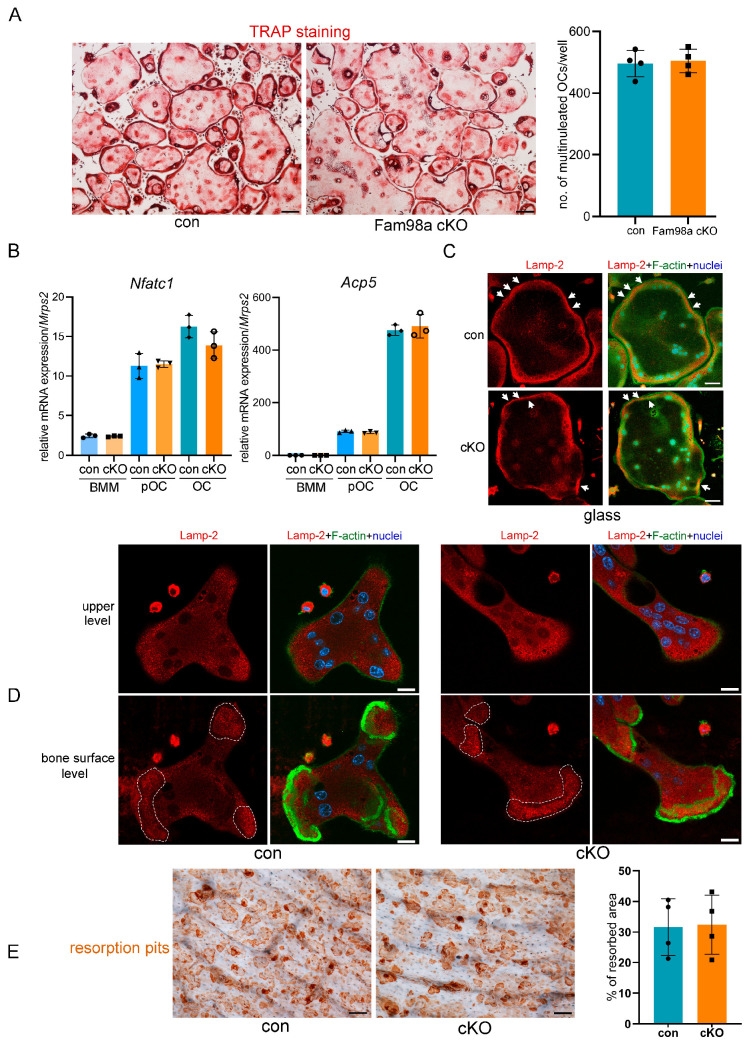
Loss of Fam98a in myeloid osteoclast precursor cells is dispensable for osteoclastogenesis and bone resorption in vitro. (**A**) TRAP staining and cell number count of osteoclasts differentiated from the BMMs isolated from control (con) and Fam98a-cKO mice. *n* = 4. Scale bar = 20 µm. (**B**) The RT-qPCR detection of mRNA expression of osteoclast marker genes, *Nfatc1* and *Acp5*, in control and Fam98a^−/−^ BMMs, pre-OCs, and mature osteoclasts. *n* = 3. (**C**) The confocal microscopic images of triple-staining of Lamp-2, F-actin, and nuclei in control and Fam98a^−/−^ osteoclasts cultured on glass coverslips. The white arrows point to the peripheral distributed Lamp-2. Scale bar = 20 µm. (**D**) The confocal microscopic images of Lamp-2 at the upper and bone surface levels in control and Fam98a^−/−^ osteoclasts cultured on bovine cortical bone chips. The white dashed lines demarcate the Lamp-2 staining at the ruffled border membrane circumscribed by actin rings. Scale bar = 20 µm. (**E**) The resorption pit staining and quantification. Scale bar = 20 µm. *n* = 4.

To unveil the impacts of loss of Fam98a in osteoclast precursors on osteoclastogenesis and bone resorption in vitro, we cultured the BMMs isolated from control and Fam98a cKO mice, respectively, with M-CSF and RANKL for 4 days on plastic culture dishes, glass coverslips, and cortical bovine bone slices. The number of TRAP^+^ multinucleated osteoclasts and the mRNA expression of *Nfatc1* and *Acp5* in Fam98a-deficient BMMs, pre-osteoclasts, and mature osteoclasts were very close and indistinguishable from those in control cells (Figure 6A,B). In contrast to our previous findings in Fam98a shRNA knockdown osteoclasts [6], genetic ablation of Fam98a in osteoclasts had no effects on the peripheral distribution of Lamp-2^+^ lysosomes in osteoclasts cultured on glass coverslips and lysosome trafficking and targeting to the ruffled border in osteoclasts cultured on bone slices (Figure 6C,D). Thereafter, Fam98a^−/−^ osteoclasts had a similar bone-resorbing capacity as compared to control osteoclasts (Figure 6E). Given that Fam98b is structurally and functionally related to Fam98a (Figure 1 and Figure 4), it is likely that Fam98b may compensate for the loss of Fam98a in osteoclasts.

**Figure 7:**
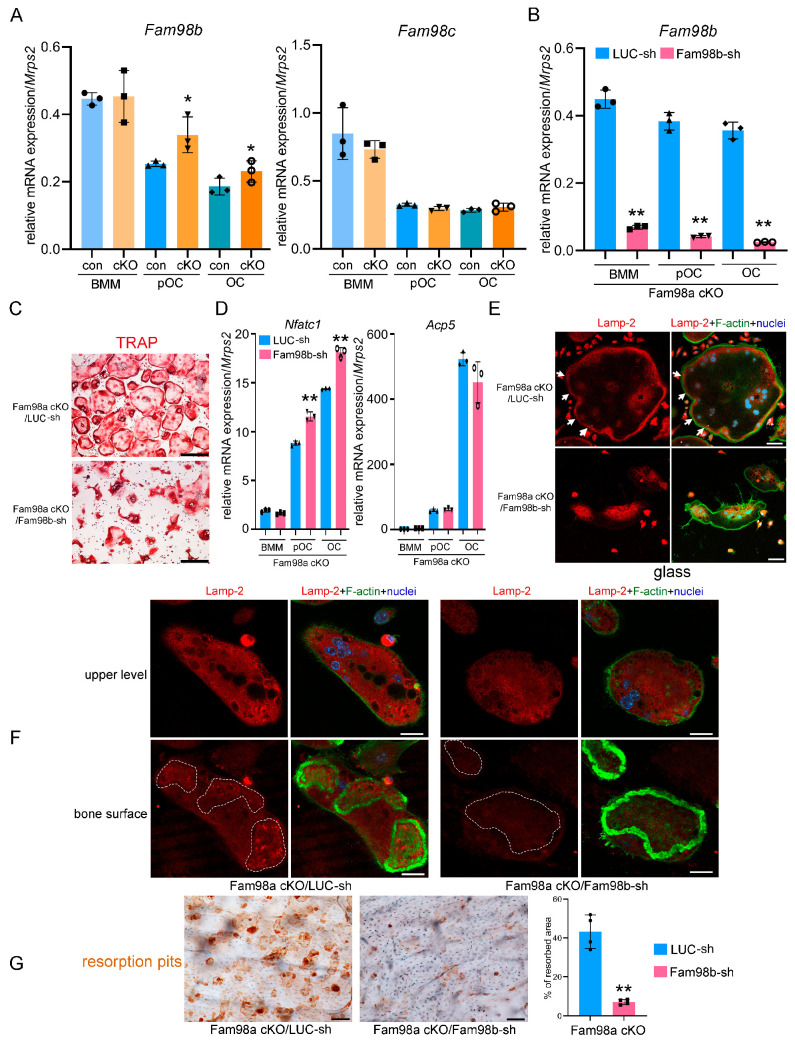
**Fam98a and Fam98b compensate for regulation of lysosome secretion and bone resorption in osteoclasts in vitro.** (**A**) The mRNA expression of *Fam98b* and *Fam98c* in control and Fam98a^−/−^ BMMs, pre-OCs, and mature OCs as detected by RT-qPCR. (**B**) The mRNA level of *Fam98b* in Fam98a^−/−^ osteoclast lineage cells transduced with the LUC-sh and Fam98b-sh. (**C**) TRAP staining of osteoclasts cultured from the LUC-sh and Fam98b-sh transduced Fam98a^−/−^ BMMs. Scale bar = 80 µm. (**D**) The mRNA expression of osteoclast marker genes, *Nfatc1* and *Acp-5*, in control and Fam98a^−/−^/Fam98b^KD^ osteoclast lineage cells. (**E**,**F**) The confocal microscopic images of triple-staining of Lamp-2, F-actin, and nuclei in control and Fam98a^−/−^/Fam98b^KD^ osteoclasts cultured on glass coverslips and bovine cortical bone chips, respectively. The white arrows in (**E**) point to the peripheral distributed Lamp-2 in control osteoclasts. Scale bar = 20 µm. The white dashed lines in (**F**) demarcate the Lamp-2 staining at the ruffled border membrane circumscribed by actin rings in control osteoclasts. Scale bar = 20 µm. (**G**) The resorption pit staining and quantification. Scale bar = 20 µm. * *p* < 0.05, ** *p* < 0.01 vs. the respective control cells analyzed by one-way ANOVA. *n* = 3–4.

To test if the lack of skeletal phenotype in Fam98a cKO mice can be explained by increased Fam98b expression, we first examined the mRNA expression of *Fam98b* and *Fam98c* in control and Fam98a^−/−^ BMMs and pre-osteoclasts and mature osteoclasts. The expression of *Fam98b* but not *Fam98c* was upregulated in Fam98a^−/−^ osteoclast precursor and mature cells compared to respective control osteoclasts (Figure 7A). Next, we transduced the Fam98a^−/−^ BMMs with lentiviruses expressing the control (LUC-sh) and Fam98b-specific (Fam98b-sh) shRNAs, respectively. The mRNA level of *Fam98b* was dramatically and significantly reduced in Fam98b-sh transduced osteoclast precursor and mature cells (Figure 7B). The osteoclastogenesis from Fam98a^−/−^/Fam98b^KD^ BMMs was comparable to the control BMMs with a slight increase in *Nfatc1* but not *Acp5* mRNA expression (Figure 7C,D) in Fam98a/b double-depleted osteoclast precursor and mature cells. However, the majority of Fam98a^−/−^/Fam98b^KD^ osteoclasts (77 ± 12%, *n* = 4) exhibited irregular morphology with an accumulation of TRAP^+^ lysosomes around nuclei (Figure 7C, lower panel) compared to Fam98b single knockdown osteoclasts (50.7 ± 8.4%, *n* = 4, in Figure 4B). The severe defects in lysosome trafficking and bone resorption in Fam98a^−/−^/Fam98b^KD^ osteoclasts were observed by Lamp-2 immunofluorescent and bone resorption pit staining (Figure 7E–G). These results postulate that Fam98a and Fam98b may compensate for each other in the regulation of lysosome secretion and bone resorption in osteoclasts in vitro and in vivo. This hypothesis warrants further investigation in the future.

## 4. Discussion

Three FAM98 family proteins (FAM98A/B/C) have been identified in humans and mice based on sharing a unique structure domain with unknown function (DUF2465). By bioinformatic profiling and structural modeling, this domain is also found in several microtubule-associated proteins, including the intraflagellar transport (IFT) complex B subunits (IFT81, IFT57, and CLUAP1), suggesting a hypothetic role of FAM98 family proteins in microtubule dynamics and/or microtubule-based transportations [25]. FAM98A and FAM98B have been shown to work in an RNA-binding complex, positively regulating RNA transcription, tRNA splicing, and RNA translation [15,16,17,18,26]. FAM98C has been identified as a candidate gene linked to ciliogenesis [19]. Nonetheless, the expression and physiological functions of FAM98A/B/C in bone cells and skeleton modeling/remodeling have not been fully elucidated and remain largely unknown.

We have previously reported that Fam98a interacts with Plekhm1 in murine osteoclasts and suppression of Fam98a expression by shRNA-mediated specific knockdown of *Fam98a* mRNA in osteoclasts attenuates osteoclast lysosome trafficking and bone resorption, indicating that FAM98A might couple lysosomes to microtubules via its binding to lysosomal adaptor protein PLEKHM1 [6]. Unexpectedly, however, we found in this study that loss of Fam98a by its genetic deletion in osteoclast myeloid precursor cells was dispensable for osteoclastogenesis and osteoclast function in vitro as well as bone modeling/remodeling in vivo. Since the knockdown of Fam98b expression by shRNA in control and Fam98a^−/−^ osteoclasts exhibited defects in lysosome trafficking and bone resorption and since Fam98b expression was higher in Fam98a^−/−^ osteoclasts than in control cells, it is likely that Fam98b compensates for the loss of function of Fam98a in osteoclasts. Fam98b retains more structural similarity with Fam98a than Fam98c, and FAM98A/B has been shown to function redundantly in colorectal cancer cells [26]. As constituted in a protein complex regulating RNA metabolism and translation, FAM98A/B may activate PLEKHM1 mRNA maturation and/or translation. Thereby, suppression of FAM98A/B may cause a decreased level of PLEKHM1, which is indispensable for lysosome trafficking and bone resorption in osteoclasts [6]. Unfortunately, there is no reliable and high-quality antibody available to detect endogenous PLEKHM1 to test this hypothesis. Alternatively, FAM98A/B may anchor lysosomes onto microtubules via its interaction with PLEKHM1 and the N-terminal conserved DUF2465 domain. Nevertheless, the underlying mechanisms by which FAM98A/B regulates osteoclast lysosome trafficking and bone resorption warrant further investigation in the future.

The mRNA level of Fam98c was the highest among the three *Fam98* genes in cultured murine osteoclast precursor and mature cells, as detected by RT-qPCR in the present study. Moreover, repression of Fam98c but not loss of Fam98a/b greatly reduced the mRNA and protein levels of the master osteoclast differentiation transcription factor Nfatc1 and dramatically inhibited osteoclastogenesis in vitro. These results indicate that FAM98C plays a critical role in osteoclast formation, at least in vitro. The exact mechanisms by which FAM98C specifically regulates osteoclast differentiation and/or survival are unknown. One possibility is that FAM98C may be involved in NFATC1 mRNA processing and translation since FAM98C has been linked to ciliogenesis [19] and the primary cilium has been recently reported to regulate osteoclastogenesis [27]. It is also likely that FAM98C regulates osteoclast differentiation through its action on the primary cilia dynamics. However, it should be noted that the important role of primary cilium in the regulation of osteoclasts has not yet been well established compared to osteoblasts and osteocytes [28,29] and requires more future research.

Although our work so far has uncovered unique roles of FAM98 family proteins in osteoclastogenesis and osteoclast bone resorption, there are several limitations of the current study. First, the detailed and comprehensive mechanisms by which FAM98A/B and FAM98C regulate lysosome secretion/bone resorption and osteoclastogenesis, respectively, have not been elucidated and need further investigations in the future. Moreover, the compensatory changes of other components of lysosome-trafficking machinery in FAM98A/B-deficient osteoclasts have not been executed and require further studies. Second, FAM98A and FAM98B have been implicated in tumorigenesis and progression. Whether and how FAM98A/B regulates lysosome secretion and degradation of extracellular matrix in cancer cells have not been conducted in this study. In addition, whether and how the downregulation of FAM98A and FAM98B plays a role in osteoclast-mediated cancer bone metastasis need studies using relevant animal models. Third, the functions of FAM98 family proteins under pathological conditions and in human disease-relevant animal models have not been carried out in this study. Such investigations will significantly improve the clinical and translational implications of targeting FAM98 family proteins as novel therapeutic strategies for the treatment of metabolic bone diseases and cancers.

## 5. Conclusions

FAM98 family proteins play distinct roles in osteoclastogenesis and osteoclast bone resorption, respectively. FAM98C plays an important role in osteoclastogenesis, whereas FAM98A and FAM98B specifically regulate osteoclast lysosome trafficking and bone resorption.

## Figures and Tables

**Table 1:** The key reagents used in this study.

Reagent Type	Designation	Source	Catalog Number	Information
Cell cultures	Alpha-MEM	MilliporeSigma, St. Louis, MO, USA	M0644-10 × 1 L	
	High glucose DMEM	MilliporeSigma, St. Louis, MO, USA	D5648-10 × 1 L	
	10× penicillin–streptomycin-L-glutamine	MilliporeSigma, St. Louis, MO, USA	G1146	
	Fetal bovine serum (FBS)	R&D Systems, Minneapolis, MN, USA		
	10× Red Blood Cell Lysis Buffer	Abcam, Cambridge, MA, USA	ab204733	
	10× Trypsin/EDTA	Thermo-Fisher Scientific, Waltham, MA, USA	15400-054	
	Puromycin	MilliporeSigma, St. Louis, MO, USA	P8833-10MG	6 µg/mL
Antibodies	Mouse monoclonal anti-Nfatc1	Santa Cruz Biotechnology, Dallas, TX, USA	sc-7294	WB 1:250
	Mouse monoclonal anti-Cathepsin K	MilliporeSigma, St. Louis, MO, USA	MAB3324	WB 1:2000
	Mouse monoclonal anti-Tubulin	MilliporeSigma, St. Louis, MO, USA	T9026	WB 1:5000
	Rat monoclonal anti-Lamp-2	Developmental Studies Hybridoma Bank, Iowa City, IA, USA	GL2A7	IF 1:200
	HRP-goat anti-mouse	Cell Signaling Technology, Danvers, MA, USA	7076	WB 1:5000
	TRITC AffiniPure donkey anti-rat	Jackson Immunoresearch, West Grove, PA, USA	712-025-150	IF 1:200
Primers	Murine *Fam98a*-exon 4 forward	IDT, Coralville, IA, USA	5′-TTGTGTCAGTTAAAGGAAAC-3′	
	Murine *Fam98a*-exon 4 backward	IDT, Coralville, IA, USA	5′-TGGGCTGGTCCCATCGGCTTC-3′	
	Murine *β-actin* gDNA forward	IDT, Coralville, IA, USA	5′-TTCGCCATGGATGACGATATC-3′	
	Murine *β-actin* gDNA backward	IDT, Coralville, IA, USA	5′-GAATACAGCCCGGGGAGCATC-3′	
	Murine *Fam98a*-flox forward	IDT, Coralville, IA, USA	5′-GAGCCCAGGTTGGCCTCAGATTCT-3′	
	Murine *Fam98a*-flox backward	IDT, Coralville, IA, USA	5′-ACTGACGCTGTCACACGTGACTCC-3′	
	Murine *Fam98a*-FAM	Thermo-Fisher Scientific, Waltham, MA, USA	Mm01223835_m1	qPCR primer
	Murine *Fam98b*-FAM	Thermo-Fisher Scientific, Waltham, MA, USA	Mm01277500_m1	qPCR primer
	Murine *Fam98c* -FAM	Thermo-Fisher Scientific, Waltham, MA, USA	Mm00503820_m1	qPCR primer
	Murine *Nfatc1*-FAM	Thermo-Fisher Scientific, Waltham, MA, USA	Mm00479445_m1	qPCR primer
	Murine *Acp5*-FAM	Thermo-Fisher Scientific, Waltham, MA, USA	Mm00475698_m1	qPCR primer
	Murine *Mrps2*	Thermo-Fisher Scientific, Waltham, MA, USA	Mm00475529_m1	qPCR primer
Plasmids	pLKO.1 Fam98b-sh	MilliporeSigma, St. Louis, MO, USA	TRCN 0000190520	lentiviral targeting vector
	pLKO.1 Fam98c-sh	MilliporeSigma, St. Louis, MO, USA	TRCN 0000192515	lentiviral targeting vector
	pLKO.1 LUC-sh	MilliporeSigma, St. Louis, MO, USA	SHC007	lentiviral targeting vector
	pMD2.G	Addgene, Watertown, MA, USA	12259	lentiviral packaging vector
	pCMV-delta R8.2	Addgene, Watertown, MA, USA	12263	lentiviral packaging vector
Compounds and solutions	Protamine sulfate	MilliporeSigma, St. Louis, MO, USA	P4020-1G	lentiviral transduction
	Opti-MEM	Thermo-Fisher Scientific, Waltham, MA, USA	11058-021	plasmid transfection
	TransIT-LT1	Mirus Bio LLC, Madison, WI, USA	MIR2300	plasmid transfection
	NaNO_2_	MilliporeSigma, St. Louis, MO, USA	S2252	TRAP staining
	NaK tartrate	MilliporeSigma, St. Louis, MO, USA	S6170	TRAP staining
	Na acetate	MilliporeSigma, St. Louis, MO, USA	S2889	TRAP staining
	Pararosaniline	MilliporeSigma, St. Louis, MO, USA	P3750	TRAP staining
	Naphthol AS-BI	MilliporeSigma, St. Louis, MO, USA	1802	TRAP staining
	RIPA buffer	MilliporeSigma, St. Louis, MO, USA	R-0278	cell lysis
	cOmplete, EDTA-free Protease Inhibitor Cocktail	MilliporeSigma, St. Louis, MO, USA	4693159001	cell lysis
	Enhanced chemiluminescent detection reagents (ECL)	MilliporeSigma, St. Louis, MO, USA	WBKLS0100	WB
	Paraformaldehyde	MilliporeSigma, St. Louis, MO, USA	P6148	cell fixation
	Saponin	MilliporeSigma, St. Louis, MO, USA	S-4521-10G	IF
	Bovine serum albumin	MilliporeSigma, St. Louis, MO, USA	BSAV-RO	IF
	Alexa Fluro-488 phalloidin	Thermo-Fisher Scientific, Waltham, MA, USA	A12379	IF
	Peroxidase-conjugated WGA (wheat germ agglutinin) lectin	MilliporeSigma, St. Louis, MO, USA	L-7017	pit staining
	3,3′-diaminobenzidine (DAB) tablets	MilliporeSigma, St. Louis, MO, USA	D-5905	pit staining
	30% H_2_O_2_	MilliporeSigma, St. Louis, MO, USA	216763	pit staining
Kits	RNeasy mini kit	Qiagen, Germantown, MD, USA	74104	RNA purification
	High-capacity cDNA RT kit	Thermo-Fisher Scientific, Waltham, MA, USA	4368813	cDNA RT
	TaqMan Gene Expression Master Mix	Thermo-Fisher Scientific, Waltham, MA, USA	4369016	qPCR reaction

## Data Availability

The data generated in this study are available from PI upon request.

## Supplementary Materials

The following supporting information can be downloaded at: https://www.mdpi.com/article/10.3390/biology14010045/s1, The uncropped film images of immunoblotting shown in Figure 3D.
