# Supplementary figures and images for: FAM98 Family Proteins Play Distinct Roles in Osteoclastogenesis and Bone Resorption
Source: Biology (Basel). 2025 Jan 9;14(1):45. doi: 10.3390/biology14010045 (PMC11762708; doi:10.3390/biology14010045)

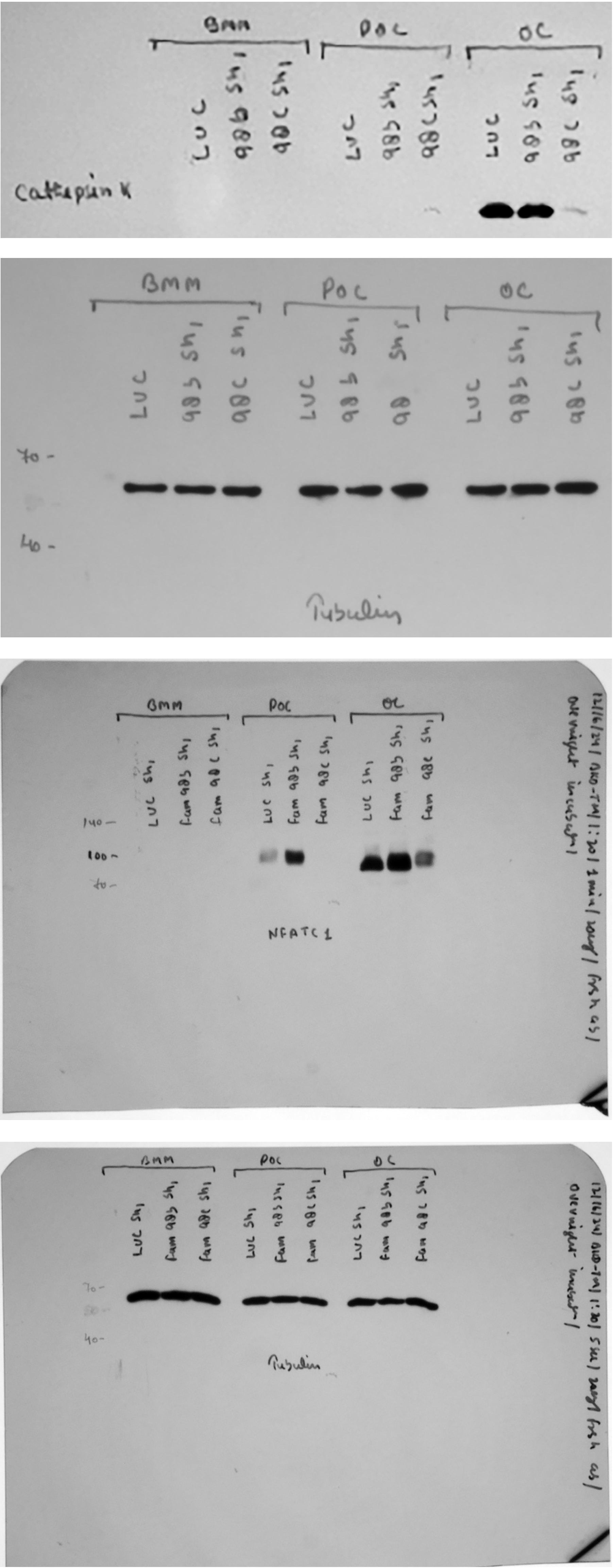

Supplement: Supplementary file 1 [file biology-14-00045-s001.zip › biology-3303032-supplementary.tif]
